# Inhaled Nitric Oxide (iNO) With a Massive Pulmonary Embolism: A Case Report

**DOI:** 10.7759/cureus.27122

**Published:** 2022-07-21

**Authors:** Mohammad Ismail, Moayad Bsooki, Badr Allehyani, Ahmed Alessa

**Affiliations:** 1 Respiratory Therapy Administration, King Abdullah Medical City, Makkah, SAU

**Keywords:** hfnc, non-invasive mechanical ventilation, pe, bipap, nitric oxide

## Abstract

Massive pulmonary embolism (PE) is a type of complication related to the migration of deep venous thrombi clot to the lungs. Massive PE is associated with a high level of morbidity and mortality due to elevated pulmonary vascular resistance that can cause right ventricular failure, cardiogenic shock, and hypoxemia. This report aims to explain to the readers the efficacy of applying inhaled nitric oxide (iNO) to patients with a massive PE. It also aims to evaluate iNO’s pulmonary vasodilator efficacy for acute PE.

## Introduction

Massive pulmonary embolism (PE) is a type of complication related to the migration of deep thrombi venous clots to the lungs. So far, there is little progress in finding an effective intervention for this major complication. Massive PE is associated with a high level of morbidity and mortality due to elevated pulmonary vascular resistance that can cause right ventricular failure, cardiogenic shock, and hypoxemia [1]. This report aims to explain to the readers the efficacy of applying inhaled nitric oxide (iNO) to patients with a massive PE. It also aims to evaluate iNO’s pulmonary vasodilator efficacy for acute PE [2]. The variables of morbidity include cardiac symptoms and the echocardiographic presence of thrombus. If iNO has no efficacy, drug therapy should be an option in cases where there is no rapid deterioration [3].

## Case presentation

**Figure 1:**
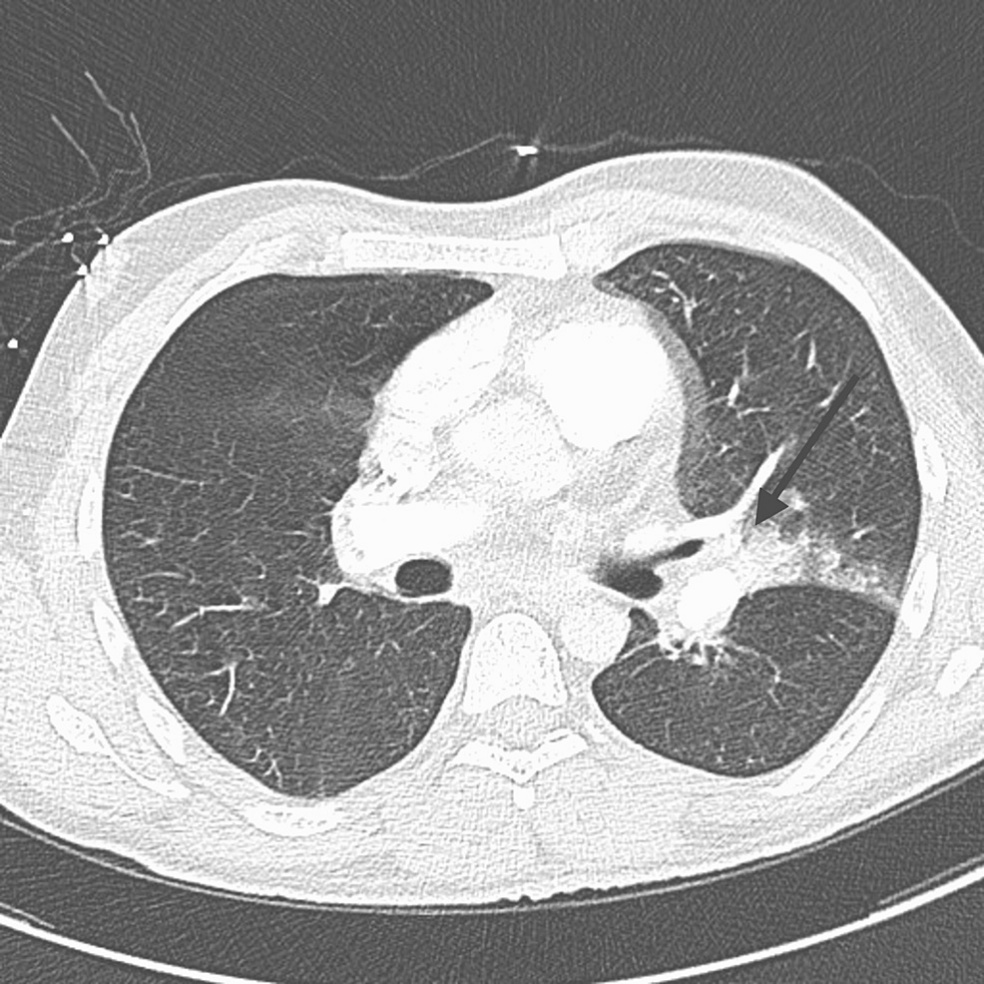
High-resolution computed tomography (HRCT) scan showed redemonstration of an extensive PE with right ventricular strain PE: pulmonary embolism

A 35-year-old man was admitted on September 6, 2021, due to massive PE and intracardiac thrombus. He was brought to the Emergency Room (ER) and his medical history was analyzed. He reported that he had taken one dose of the coronavirus disease 2019 (COVID-19) vaccine (AstraZeneca) around two months back. The patient complained about major pain from dyspnea that gradually increased and became worse. In addition, the patient was also complaining of severe chest pain mainly on the left side, with increasing respiration and chest movement. The initial assessment (Table 1) showed that the patient was conscious (Glasgow Coma Scale (GCS) 15/15) and recorded tachycardia (heart rate (HR) 112) with 109/80 mmHg blood pressure (BP). His breath sound was bronchial but tachypneic (respiratory rate (RR) 25 breaths per minute (BPM)). His peripheral capillary oxygen saturation (SpO2) was 99% on 6 liters per minute (LPM) simple face mask, his abdomen was soft, and his urine output was normal. The echocardiogram (ECHO) examination showed that the patient’s right ventricle (RV) was moderately dilated and depressed in function. His right atrium (RA) was dilated and there was a large irregular shape mobile mass in RA protruding into RV. It was attached to the upper side of the RA. There was also a mild to moderate tricuspid regurgitation. The right ventricular systolic pressure (RVSP) was 50-60 mmHg. The Doppler ultrasound (USG) showed right-sided deep vein thrombosis (DVT). The computed tomography (CT) scan for PE in Figure 1 showed redemonstrations of an extensive PE with right ventricular strain. Mild improvement of the filling defect in the left lung showed a resolution of the non-obstructive filling defect previously seen in the left main pulmonary artery. The interval development of air space lesion in the posterior segment of the left upper lobe can be related to early infarction versus infection. Further diagnosis revealed WBC 15.66, hemoglobin 16.0 gm/dl, hematocrit (HCT) 51.1%, platelets 91 10^9^/L, and neutrophils 12.6.

**Table 1:** Laboratory results and vital signs BPM: beats per minute

Lab Tests and Vital Signs	Normal Value	Admission, September 6, 2021	September 7, 2021	September 8, 2021	September 9, 2021	September 10, 2021	September 15, 2021
Heart Rate (HR)	60-100 BPM	112	115-125	108-115	96	100	81
Respiratory Rate (RR)	12-20 BPM	25	30	20-25	18-20	15-22	12-15
Creatinine (Cr)	0.74 - 1.35 mg/dL	1.56	1.18	0.85	0.99	0.75	0.84
Blood Urea Nitrogen (Bun)	6 – 25 mg/dL	30.54	21.45	15	15.67	16.21	20.07
Glasgow Coma Scale (GCS)	15/15	15/15	15/15	15/15	15/15	15/15	15/15
Urine Output ml/hr		140-50	100-200	200-300	100-200	150-250	200-250
Blood Pressure (BP)		109/80	112/78	124/67	110/68	125/70	125/73

Treatment

On September 7, 2021, the patient was admitted to ICU. He was alert and conscious (GCS 15/15) and hemodynamically stable with tachycardia (HR 115-125). He started to be tachypneic (RR 30) with increasing oxygen requirements. Thus, he was connected to the high flow nasal cannula (HFNC) with a flow of 45LPM and a fraction of inspired oxygen (FiO2) of 90%. In taking a liquid diet with lax abdominal ingestion, his creatinine (Cr) improved (1.56 > 1.18) as well as his blood urea nitrogen (BUN) (30.54 > 21/45) and he had good urine output (100-200 ml/hr).

The patient received alteplase, ceftriaxone, azithromycin, and aspirin, and was started heparin infusion. On September 8, 2021, he was alert, anxious, and conscious (GCS 15/15). His hemodynamic was stable with HR 108-115 BPM and BP 124/67 mm/Hg. ECHO report showed persistence of RA thrombus with severe pulmonary hypertension. The patient became more tachypneic and not tolerant to HFNC. He was then connected to non-invasive ventilation (NIV) bilevel positive airway pressure (BiPAP) inspiratory positive airway pressure (IPAP) 10, and expiratory positive airway pressure (EPAP) 6 on 60% FiO2, with 20 parts per million (PPM) iNO due to pulmonary hypertension.

A follow-up ECHO report on September 8, 2021, showed the left ventricle was normal in size. Left ventricular systolic function was normal and his flattened septum was consistent with RV pressure/volume overload. However, the RV was severely dilated and there was hypokinesis of RV free wall sparing the apex with MacCallan, a sign of pulmonary embolism. The right atrium was dilated and there was a large, irregularly-shaped mobile mass in RA protruding into RV (33* 15 mm), which appeared slightly decreased in size as compared to the previous study. There was mild to moderate tricuspid regurgitation. Right ventricular systolic pressure was elevated at > 60mmHg. Catheter-directed thrombolysis was inserted. Bilateral femoral vein placed catheters were removed by the interventional radiology (IR) team and bilateral femoral vein sheaths including one inserted in the left femoral vein by IR in place.

On September 9, 2021, there was post-catheter-directed thrombolysis with active bleeding. The patient was conscious and alert. His hemodynamic was stable with HR 96 BPM and BP 110/68 mm/Hg. He was still connected to NIV with BiPAP 10/6 at 50%, RR was 18-20 BPM and SpO2 was 96%. iNO was weaned by 2PPM every two hours. His abdomen was soft with adequate urine output. An inferior vena cava (IVC) filter was inserted, and femoral vein sheaths were removed after IVC placement. His WBC was 10.37, hemoglobin 13.3, HCT 41.6%, and platelets 35. On September 10, 2021, post-IVC filter insertion was conducted, and the patient was conscious (GCS 15/15). He was hemodynamically stable (HR 100 BPM) with BP 125/70. NIV and iNO were stopped and the patient had HFNC with a flow of 40LPM and FiO2 of 40%. He had no signs of distress, his abdomen was soft, and his urine output was adequate. The patient received IV Immunoglobulin therapy. CT PE study on September 12, 2021, showed mild improvement of bilateral extensive PE and stable features of right ventricular strain and pulmonary hypertension. This is a significant improvement of the previously described left upper lobe and right lower lobe air space opacities, consistent with an improved parenchymal thromboembolic burden. New peripheral ground-glass opacities were seen at the bilateral upper lobes that could relate to thromboembolic disease.

On September 13, 2021, the patient was conscious, oriented, and hemodynamically stable with a nasal cannula at 4LPM at SpO2 98%. He developed severe thrombocytopenia with thrombosis. The HIT study was negative but vaccine-induced immune thrombotic thrombocytopenia (VITT) was possible. He received bivalirudin as a replacement for heparin. On September 15, 2021, the patient was conscious, oriented, and his hemodynamic was stable upon bivalirudin infusion. His abdomen was soft with Cr 0.84, BUN 20.07, and he had a good urine output. RT Doppler USG on September 19, 2021, showed that the known deep venous thrombus found in the proximal right superficial femoral vein down to the popliteal vein showed a partial recanalization. Otherwise, there was no interval change.

## Discussion

PE is generally suspected in all patients who have worsening dyspnea, chest pain, or sustained hypotension without an alternative obvious cause [4]. During diagnosis, it was found that acute PE is dependent on hemodynamic instability. In severe cases, doctors often use the findings of bedside echocardiography and apply a thrombolytic treatment without first conducting a diagnosis in nuclear imaging. PE is the third most common cause of death from cardiovascular disease after a heart attack and stroke [5]. PE remains poorly understood. According to studies, anticoagulant treatment revealed an effectiveness of 96% for the prevention of PE in high-risk patients. On the other hand, patients with PE who were subject to systematic perfusion lung scans were not specific indicators of recurrent PE [6].

Primary therapy is an alternative for PE patients who experience ventricular dysfunction on ECHO [7]. Increasing the pulmonary flow of blood through isoproterenol infusion or decreasing it through a partial bypass of the heart’s right side can minimally alter the pulmonary artery [8]. RV failure reversed and hyperemia of the right-side coronary flow was restored. This condition demonstrates that ischemia is the major cause of acute hypertension RV failure [9]. Rates of clinical outcomes such as death and recurrence vary widely among trials [10]. Sequelae occurring after venous thromboembolism include chronic thromboembolic pulmonary hypertension and post-thrombotic syndrome [5]. The main symptoms of PE are dyspnea and increasing work of breathing (WOB). Bi-Level Positive Airway Pressure (BiPAP) can resolve dyspnea and decrease WOB. Some patients require intubation if BiPAP did not make any effect. PE can lead to pulmonary hypertension that causes hypoxia.

iNO is considered as a gas that could cause selective vasodilation of ventilated lung regions, thereby reducing pulmonary hypertension and improving gas exchange [11,12]. iNO is a toxic gas that produces methemoglobins and nitrogen dioxide (NO2) [13]. In our patient, iNO with BiPAP was the main cause of decreasing WOB, dyspnea, and desaturation to avoid intubation. iNO was given for two days, starting with 20 ppm. After one day, weaning was started by decreasing 2ppm every two hours. To our knowledge, very few cases have been reported in the medical literature where iNO can delay intubation and give effective results. Current management strategies for acute major PE are largely dependent on the degree of hemodynamic instability at presentation. In the presence of severe hemodynamic compromise, physicians often rely on the findings of bedside echocardiography and proceed to thrombolytic treatment without seeking further diagnostic certainty in nuclear imaging or angiographic studies [14].

## Conclusions

To conclude, PE is a very common cause of death from cardiovascular disease, similar to heart attack and stroke. However, pulmonary hypertension has unknown complications. In this case, iNO gave a good result when connected with BiPAP. The gas nitric oxide (NO) is an endothelium-derived relaxing chemical, inactivated by combining heme with hemoglobin. Inhaling it properly as performed by doctors can delay intubation, give better outcomes, and decrease pulmonary hypertension. However, since iNO produces methemoglobins and NO2, which are toxic components, they need to be observed closely. Therefore, more research is needed to provide scientific data about this case and to try these methods. According to studies, the pulmonary vascular resistance of patients with pulmonary hypertension fell significantly after iNO and after taking prostacyclin. Likewise, the pulmonary vascular resistance also fell significantly in cardiac patients after inhaling NO. Therefore, iNO is an effective pulmonary vasodilator.
